# Role of molecular biomarkers in glioma resection: a systematic review

**DOI:** 10.1186/s41016-020-00198-x

**Published:** 2020-05-18

**Authors:** Lianwang Li, Yinyan Wang, Yiming Li, Shengyu Fang, Tao Jiang

**Affiliations:** 1 Beijing Neurosurgical Institute, Capital Medical University, No. 119 South 4th Ring West Road, Fengtai District, Beijing, 10070 China; 2 Department of Neurosurgery, Beijing Tiantan Hospital, Capital Medical University, No. 119 South 4th Ring West Road, Fengtai District, Beijing, 10070 China

**Keywords:** Glioma, Molecular biomarkers, Guidance, Resection

## Abstract

New discoveries based on genetic and epigenetic evidence have significantly expanded the understanding of diffuse gliomas. Molecular biomarkers detected in diffuse gliomas are not only potential targets for radiotherapy, chemotherapy, and immunotherapy, but are also able to guide surgical treatment. Previous studies have suggested that the optimal extent of resection of diffuse gliomas varies according to the expression of specific molecular biomarkers. However, the specific guiding role of these biomarkers in the resection of diffuse gliomas has not been systemically analyzed. This review summarizes several critical molecular biomarkers of tumorigenesis and progression in diffuse gliomas and discusses different strategies of tumor resection in the context of varying genetic expression. With ongoing study and advances in technology, molecular biomarkers will play a more important role in glioma resection and maximize the survival benefit from surgery for diffuse gliomas.

## Main text

### Introduction

Histologically, diffuse gliomas originate from aberrant neural progenitor stem cells of the central nervous system and composed of either astrocytes or oligodendrocytes. Although the morphology-based classification system from the World Health Organization (WHO) in 2007 is highly significant for the characterization of diffuse gliomas, it is unable to explain the evident differences in prognosis of patients with the same histopathology [1, 2]. Molecular biology studies have provided new insights into the oncogenesis and progression of diffuse gliomas and have prompted neurologists to reconsider the biological characteristics of diffuse gliomas. Hence, in 2016, the WHO updated the classification of diffuse gliomas, with the new classification integrating traditional histological and new molecular biological features, which has effectively improved the consistency of prognosis of diffuse gliomas [3].

The goal of traditional treatment strategies for diffuse gliomas was to achieve maximum resection in the context of protecting neurological and functional integrity, and further adjuvant radiotherapy and/or chemotherapy is applied according to the extent of resection (EOR) and histopathology [4]. The degree of EOR is determined according to tumor volume on preoperative and postoperative magnetic resonance imaging (MRI). According to previous studies, gross total resection (GTR) of a non-enhancing tumor is defined as the absence of high-intensity lesion(s) on postoperative T2-weighted/fluid attenuation inversion recovery sequence. GTR of enhanced tumor(s) is defined as the absence of postoperative enhancement. Subtotal resection is defined as the presence of < 10% residual lesion on postoperative imaging [5, 6].

Multiple retrospective studies and large meta-analyses have demonstrated that, regardless of low- or high-grade, primary or recurrent, greater EOR can significantly prolong both progression-free survival (PFS) and overall survival (OS) of diffuse gliomas compared with partial resection or biopsy [7–9]. However, most of these studies were based on histopathological diagnoses and did not consider molecular biology. Molecular biomarkers are related to the malignancy of diffuse gliomas and have prognostic value in predicting the efficiency of radiotherapy and chemotherapy [10]. Recent studies have also confirmed the interaction effect between molecular biomarkers and EOR in diffuse gliomas [5, 6, 11–14]. For some molecular pathology types, GTR or even supra-total resection was essential [5], while for the others, GTR had no survival benefit but increased the risk for operative complication(s) [6, 11]. To date, the impact of molecular biomarkers for guiding glioma resection has not been systematically addressed. Accordingly, we retrospectively reviewed the literature pertaining to significant molecular biomarkers in the oncogenesis and progression of diffuse gliomas, as well as their guiding role in surgical treatment to provide comprehensive information on which to base clinical and preclinical research (Table 1).

**Table 1:** Summary of previous studies and recommended level according to the Oxford Centre for Evidence-based Medicine Levels of Evidence

Authors [reference]	Molecular marker	WHO grade	Patient number	Surgical guidance	Level
Wijnenga et al. [13]	IDH, 1p/19q	2	228	IDHmt, 1p/19q non-codel: GTR; IDHmt, 1p/19q codel: GTR unnecessary	3b
Patel et al. [12]	IDH	2	74	IDHmt: no correlation; IDHwt: the more the better	3b
Delev et al. [14]	IDH, 1p/19q	2 and 3	299	IDHmt, 1p/19q non-codel: GTR; IDHmt, 1p/19q codel: GTR unnecessary; IDHwt: the more the better	3b
Ding et al. [15]	IDH, 1p/19q	2, 3 and 4	1600	IDHmt, 1p/19q non-codel: GTR; IDHmt, 1p/19q codel: GTR unnecessary;	3b
Koriyama et al. [5]	IDH, ATRX, p53	2 and 3	–	IDHmt, loss of ATRX nuclear expression and p53 high expression: GTR; IDHmt, ATRX nuclear expression and p53 low expression: functional region > 90% resection, non-functional region-GTR; IDHwt: functional region-GTR, non-functional region-supra-total resection.	5
Kawaguchi et al. [6]	IDH, 1p/19q	3	124	IDHmt, 1p/19q non-codel: GTR; IDHmt, 1p/19q codel: GTR unnecessary; IDHwt: GTR unnecessary	3b
Beiko et al. [11]	IDH	3 and 4	335	IDHmt, 1p/19q non-codel: enhancing and non-enhancing tumor resection; IDHwt: enhancing tumor resection	3b
Sharma et al. [16]	MGMT	4	233	MGMT promoter methylation or unmethylation: enhancing tumor resection ≥ 86%	3b
Sayeed et al. [17]	MGMT	4	63	MGMT promoter methylation: EOR ≥ 95%	3b
Gessler et al. [18]	IDH, MGMT	4	175	IDHwt: GTR, no matter what MGMT promoter expression	3b
Fontana et al. [19]	EGFR	GBM cell line	–	EGFRvIII alone or with EGFR overexpression decrease 5-ALA-induced fluorescence	5
Yue et al. [20]	EGFR v III	GBM bearing mice	–	EGFRvIII nanoprobe assists in determining tumor boundaries	5
Munthe et al. [21]	CD133	2, 3, and 4	26	CD133 expressed in tumor margin cells	5
Cordier et al. [22]	1p/19q	2	200	Higher EOR associated with 1p/19q non- or single deletion	3b
Paldor et al. [23]	Ki-67	4	223	GBM in the frontal lobe show higher Ki-67 index, early treatment is recommended	3b

*Abbreviations*: *EOR* extent of resection, *GBM* glioblastoma, *GTR* gross total resection, *IDH* isocitrate dehydrogenase, *IDHmt* IDH mutation, *IDHwt* IDH wild-type, *1p/19q codel* 1p/19q codeletion, *1p/19q non-codel* 1p/19q non-codeletion, *MGMT* O6-methylguanine-DNA methyltransferase

### Isocitrate dehydrogenase

Isocitrate dehydrogenase (IDH) is an enzyme participating in the tricarboxylic acid cycle. IDH 1 and IDH 2 are two subtypes of IDH and responsible for converting isocitrate to alpha-ketoglutarate in the cytoplasm and mitochondria, thereby influencing the metabolic process. Studies have confirmed that, although IDH mutation occurs in the early stages of glioma formation and may affect DNA demethylation and lead to tumorigenesis, the exact mechanism remains unclear [24]. IDH mutation is often associated with p53 mutation, 1p/19q codeletion, or alpha-thalassemia/mental retardation syndrome X-linked (ATRX) mutation and commonly occurs in low-grade gliomas and secondary glioblastomas (GBMs), but are rare in primary GBMs [25–27].

In patients with diffuse astrocytoma, the effect of surgical resection on prognosis is different according to IDH expression and pathological grade. A previous study involving 228 adults with supra-tentorial low-grade gliomas (WHO grade 2) found that postoperative tumor volume exerted a negative effect on OS. The impact was more evident in astrocytoma with IDH mutations, in which any tumor residual will significantly reduce OS. Therefore, maximal resection in the primary operation is crucial, and a “second-look” operation may be necessary to ensure total resection for IDH-mutant tumors, if it can be performed safely [13]. Other studies have also found that increasing EOR can significantly prolong OS for patients with WHO grade 2 diffuse astrocytoma, and extensive removal (supra-total resection) is worthwhile for tumors of IDH wild-type in non-functional regions [5, 12, 28].

Regarding anaplastic diffuse astrocytoma, a study analyzed the relationship between EOR and prognosis and revealed that GTR can significantly prolong OS of patients with IDH-mutant anaplastic diffuse astrocytoma but not wild-type [6]. Similarly, another study found that EOR affected the prognosis of patients with malignant diffuse astrocytoma (WHO grade 3/4) differently according to IDH mutation expression. The authors revealed that residual enhancing tissue decreases OS in those with malignant diffuse astrocytoma of IDH wild-type, while residual either enhancing or non-enhancing tissue decreases OS of malignant diffuse astrocytoma with IDH mutation [11].

**Fig. 1:**
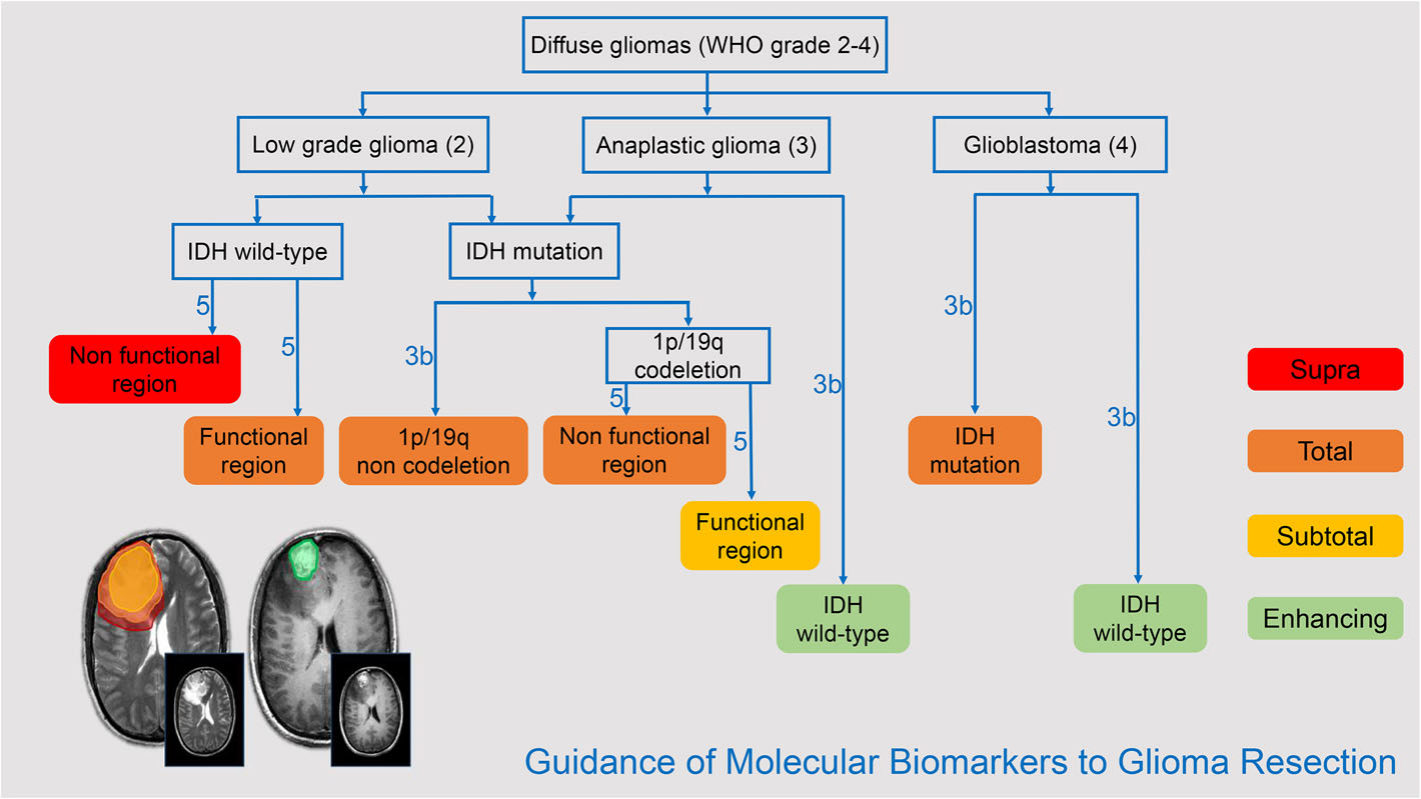
Guidance of molecular biomarkers to glioma resection. The figure shows the recommended extent of resection for diffuse gliomas according to pathological grade and molecular biomarkers. The axial T2-weighted image and contrast-enhanced T1-weighted image presented four levels of EOR as follows: red, supra-total resection; orange, total resection; yellow, subtotal resection; and green, enhancing tissue resection. Supra-total resection is recommended for low-grade diffuse gliomas with IDH wild-type in the non-functional region; subtotal resection is recommended for diffuse gliomas with IDH mutation and 1p/19q codeletion in the functional region; enhancing tissue resection is recommended for anaplastic gliomas and glioblastomas with IDH wild-type; total resection is recommended for the remaining subtypes of diffuse gliomas. The recommended level is determined according to the Oxford Centre for Evidence-based Medicine Levels of Evidence

On reviewing the existing literature, we conclude that radical surgical treatment is beneficial for patients with diffuse astrocytoma. Even if the tumor is located in a functional area, it is worthwhile to realize total resection at the cost of few partially unessential function because of the brain plasticity. Relatively conservative surgical treatment is recommended only for patients with IDH wild-type malignant diffuse astrocytomas (Fig. 1).

### 1p/19q

1p/19q chromosome codeletion results from a balanced translocation of the short arm of chromosome 1 and the centromere region of chromosome 19 and leads to the subsequent deletion of other chromosome arms [29]. It commonly coincides with IDH mutation, which corresponds to the diagnosis of oligodendroglioma according to the 2016 WHO classification of tumors of the central nervous system [3]. 1p/19q chromosome codeletion results in the mutation of the *FUBP1* and *CIC* genes, which promote tumorigenesis and cell proliferation [30, 31]. In addition, 1p/19q chromosome codeletion can improve the treatment effect of chemotherapy drugs such as procarbazine, lomustine, and vincristine (i.e., PCV) and temozolomide (TMZ), which further results in prognostic improvement [32, 33].

GTR is not always recommended for diffuse gliomas with IDH mutation and 1p/19q codeletion, especially for those located in the eloquent area. A previous study demonstrated that, in low-grade diffuse gliomas with IDH mutation and 1p/19q codeletion, GTR would not significantly prolong OS [13]. Compared with IDH-mutant astrocytoma, a small residue in low-grade diffuse glioma with IDH mutation and 1p/19q codeletion is acceptable for its slight impact on OS, which is in accordance with previous studies [15, 34]. Koriyama et al. suggested that EOR affects the prognosis of lower-grade diffuse gliomas with IDH mutation and 1p/19q codeletion (WHO grade 2/3), and GTR should be recommended if the tumor is located in the non-eloquent area. However, if the tumor is located in the eloquent area, GTR would not be necessary. Removing > 90% of tumor volumes on the basis of ensuring the integrity of neurological function is advisable [5]. Regarding anaplastic gliomas with IDH mutation and 1p/19q codeletion (WHO grade 3), GTR also has no significant survival benefit compared with partial resection, which may be associated with sensitivity to postoperative chemotherapy [6]. In summary, surgical treatment for diffuse gliomas with IDH mutation and 1p/19q codeletion should consider the location of the tumor and comprehensively protect function. It is inadvisable to achieve total resection at the expense of function impairment (Fig. 1).

### O6-methylguanine-DNA methyltransferase

O6-methylguanine-DNA methyltransferase (MGMT) is a DNA damage repair protein that removes guanine alkylation and prevents apoptosis [35, 36]. The methylation of promoters in CpG islands regulates the expression of MGMT, which further leads to reduced DNA alkylation repair efficiency and increases response to TMZ therapy, and ultimately prolongs PFS and OS in patients with high-grade diffuse gliomas [37, 38]. Approximately 40% of IDH wild-type GBMs and 80% of low-grade diffuse gliomas accompany MGMT promoter methylation [39]. Although MGMT promoter methylation has not been used to classify diffuse gliomas, many studies have revealed its significant influence on prognosis and treatment response.

MGMT promoter methylation helps to improve EOR. In MGMT promoter methylated GBMs, intraoperative ultrasound, intraoperative MRI, and 5-aminolevulinic acid (5-ALA) fluorescence staining can more accurately determine tumor boundaries, reduce the false-negative rate, and, ultimately, improve EOR [40]. Sharma et al. further investigated the relationship between EOR and MGMT promoter methylation in GBMs. Their study found that greater EOR of enhancing tumor tissues can significantly prolong PFS and OS in MGMT promoter methylated GBMs but can only prolong OS in MGMT promoter unmethylated GBMs [16]. The reason may be that MGMT promoter methylated GBMs are more sensitive to alkylating agents, and the application of TMZ can effectively prolong the time for tumor recurrence [41]. Based on 175 cases of IDH wild-type GBMs, Gessler et al. found that MGMT promoter methylated was a prognostic factor for improved OS in GBMs with complete removal of enhancing tissue [18]. Another study further reported that the survival benefit of MGMT promoter methylation was only evident when EOR was ≥ 95% [17]. Hence, greater EOR of enhancing tissue is beneficial in GBMs regardless of the expression of MGMT promoter. GTR is more expectable to be achieved in MGMT promoter methylated GBMs.

### Epidermal growth factor receptor variant III

Epidermal growth factor receptor (EGFR) is a tyrosine kinase, which is commonly activated or overexpressed in malignant astrocytoma, with the most common form of recombinant amplification being the EGFR variant type III (EGFRvIII) [42]. EGFRvIII sustainably activates the P13K/Akt pathway independent of specific receptor binding, thereby promoting GBM cell proliferation, reducing apoptosis, and increasing angiogenesis and invasiveness [43, 44]. EGFR amplification is primarily detected using fluorescence in situ hybridization. Additionally, EGFRvIII can be detected using immunohistochemistry or reverse transcription polymerase chain reaction [45]. Furthermore, EGFRvIII is a tumor-specific receptor that is expressed only on the surface of GBMs (20–30%) or other tumor cells, but not on the surface of normal cells [42, 46].

In the past few years, 5-ALA has been established as an effective intraoperative tool to increase EOR in high-grade gliomas [47]. Fontana et al. found that EGFRvIII activated heme oxygenase-1, thereby reducing the 5-ALA-induced fluorescence staining effect in GBMs [19]. This study explained the low or absent degree of 5-ALA-induced fluorescence staining in some GBMs. More importantly, this finding reminds neurosurgeons not to rely solely on 5-ALA fluorescence staining to determine tumor boundaries. In addition, Yue et al. developed a targeted nanoprobe for EGFRvIII that combines preoperative MRI and intraoperative surface-enhanced Raman scattering imaging to determine the boundaries of GBMs to guide surgical resection. The nanoprobe overcomes navigational bias caused by brain shift and accurately divides the borders of GBMs, which is expected to improve EOR and prognosis of GBMs with the EGFRvIII deletion mutation [20]. Although studies investigating EGFRvIII remain in the in vitro or animal experimentation stages, the potential impact on surgery suggests promise for further research. Other fluorescent staining reagents or techniques basing on EGFRvIII may play an increasing role in identifying the boundary and assisting the resection of diffuse gliomas.

### Other molecular markers

p53 is one of the most well-known tumor suppressor proteins to date, involving virtually all cancers, including gliomas. TP53 gene mutation involves multiple regulatory factors and is believed to promote malignant progression of low-grade glioma [48]. ATRX is a DNA helicase involved in chromatin remodeling. Loss of ATRX can result in telomerase phenotype prolongation and subsequently induces more aggressive proliferation of tumor cells [49, 50]. Currently, there are no specific treatments for these two gene mutations; however, ATRX loss, TP53 gene mutation, and IDH mutation usually occur simultaneously and are mutually exclusive from 1p/19q codeletion [51, 52]. Therefore, Koriyama et al. proposed the use of rapid immunohistochemistry to detect the expression of these two genes for further prediction of 1p/19q codeletion (accuracy = 80%) and then combined this with IDH mutation to guide surgery [5].

CD133 (also known as AC133) is a membrane-bound glycoprotein that may be involved in cell differentiation and epithelial to mesenchymal transition and is a marker of human neural stem cells [53]. A previous study revealed that marginal cells of glioma may be associated with CD133 expression [21]. Although the expression level is lower than that of the tumor core region, the expression of this stem cell marker may be related to postoperative recurrence and distant metastasis. Hence, more extensive resection or radically postoperative adjuvant therapy is beneficial to survival outcomes [54]. Ki-67 is a common cell proliferation marker. Duffau et al. found that low-grade diffuse gliomas with a higher Ki-67 index are more likely to achieve GTR [22]. In addition, GBMs in the frontal lobe or invading the bilateral cerebral hemispheres have a higher Ki-67 index, and early operation is beneficial [23]. Although the number of studies on these biomarkers remains relatively small, the underlying guiding effects in surgery are of great value.

In addition to the molecular markers mentioned above, there are many other molecular variants associated with diffuse gliomas. Further study is necessary to explore the relationship between molecular biomarkers and tumor resection to improve therapeutic effects and patient prognosis.

### Detection and prediction of molecular biomarkers

Based on the evidence mentioned above, molecular biology is, in fact, able to guide tumor resection to improve survival outcomes of patients with diffuse gliomas. Hence, preoperative prediction and intraoperative detection of molecular biomarkers are critical for glioma surgery. Several techniques have been applied to acquire both histological and molecular characteristics intraoperatively, including rapid frozen section, mass spectrometry, and real-time polymerase chain reaction, among others [55, 56]. Except for these pathological examination methods, molecular nanoprobes, as well as other fluorescence staining techniques based on molecular biomarkers, can also help neurosurgeons obtain valuable information regarding the expression of molecular biomarkers intraoperatively and guide tumor resection. However, although popularized, these techniques are time consuming [57]. Additionally, these examinations are based on craniotomy or biopsy, which cannot preoperatively guide or inform the surgical strategy.

MRI yields a significant amount of information about tumors. Recently, radiomics has emerged with the development of image feature extraction and machine learning techniques. Radiomics can automatically extract high-throughput and high-dimensional image features from medical imaging data aimed at obtaining more objective, quantitative, and invisible tumor features. Subsequently, these features could be combined with the results of molecular detection methods to establish prediction models to achieve accurate prediction before surgery [58, 59]. Based on a radiomics technique, many predictive models for different molecular biomarkers have been constructed and demonstrated high accuracy [60–65]. To perform a customized surgical treatment and achieve an optimal EOR, we developed a preoperative biomarker-predicting system based on radiomics and machine learning technology. The status of IDH, ATRX, Ki-67, EGFR, and VEGF were predicted non-invasively based on preoperative MRI [63–67]. The associated radiological features with 1p/19q codeletion were also revealed [68]. For diffuse gliomas with possibly IDH mutation and 1p/19q codeletion, functional protection should be highly considered when tumor is involving eloquent brain regions. We also found that diffuse gliomas with IDH mutation are more likely to be totally removed than those with IDH wild-type. Although the application remains initial and the “gold standard” diagnosis of molecular biomarker still depends on pathological examination, the application of these preoperative, non-invasive prediction methods represents a major trend in precision medicine in the future.

In summary, both preoperative and intraoperative techniques, including radiomics, real-time polymerase chain reaction, gap-enhanced Raman tags, molecular nanoprobes, and other fluorescence staining techniques based on molecular biomarkers, can provide valuable evidence of molecular biomarker expression and tumor margins, which could further affect the design and implementation of surgical strategies to treat diffuse gliomas. The development and application of these new techniques could establish a foundation for guiding surgical strategies with molecular biomarkers and further the progress of individualized medicine.

### Limitations

The main limitation of the present literature review was that all studies were retrospectively designed. This was inevitable because no randomized controlled clinical trials have been conducted. Additionally, the number of studies that focused on the correlation between molecular biomarkers and EOR is limited, especially when taking functional area into consideration. Thus, the level of evidence for EOR recommendations based on molecular biomarkers is relatively low.

### Conclusions

The current study revealed that molecular biomarkers can inform the design of surgical strategies for diffuse gliomas. In the era of precision medicine, individualized rather than “one-size-fits-all” operative strategies developed by comprehensive consideration of functional status and molecular biological characteristics can maximize the survival benefit to patients, which would represent a major trend in the surgical treatment of diffuse gliomas in the future.

## Data Availability

Not applicable.

## Abbreviations

ATRX: Alpha-thalassemia/mental retardation syndrome X-linked mutation
EGFR: Epidermal growth factor receptor
EOR: Extent of resection
GBM: Glioblastoma
GTR: Gross total resection
IDH: Isocitrate dehydrogenase
MGMT: O6-methylguanine-DNA methyltransferase
MRI: Magnetic resonance imaging
OS: Overall survival
PFS: Progression-free survival
TMZ: Temozolomide
WHO: World Health Organization
